# Association between Plasma HMGB-1 and Silicosis: A Case-Control Study

**DOI:** 10.3390/ijms19124043

**Published:** 2018-12-14

**Authors:** Jixuan Ma, Yun Zhou, Wei Li, Lili Xiao, Meng Yang, Qiyou Tan, Yiju Xu, Weihong Chen

**Affiliations:** 1 Department of Occupational & Environmental Health, School of Public Health, Tongji Medical College, Huazhong University of Science and Technology, Wuhan 430030, China; majixuan@hust.edu.cn (J.M.); yunz@hust.edu.cn (Y.Z.); d201781176@hust.edu.cn (W.L.); lilixiao@hust.edu.cn (L.X.); d201781175@hust.edu.cn (M.Y.); qiyoutan@hust.edu.cn (Q.T.); m201878387@hust.edu.cn (Y.X.); 2 Key Laboratory of Environment and Health, Ministry of Education & Ministry of Environmental Protection, and State Key Laboratory of Environmental Health (Incubating), School of Public Health, Tongji Medical College, Huazhong University of Science and Technology, Wuhan 430030, China

**Keywords:** biomarker, high mobility group box-1, silicosis

## Abstract

High-mobility group box-1 (HMGB-1) has been associated with fibrotic diseases. However, the role of HMGB-1 in silicosis is still uncertain. In this study, we conducted a case-control study involving 74 patients with silicosis and 107 age/gender-matched healthy controls in China. An Enzyme-linked immunosorbent assay (ELISA) was used to examine the concentrations of plasma HMGB-1 among all subjects. A logistic regression model and receiver operating characteristic curve (ROC) analysis were performed to assess the relationships between HMGB-1 and silicosis. We observed that plasma HMGB-1 concentrations were significantly increased in silicosis patients when compared with healthy controls (*p* < 0.05). Each 1 ng/mL increase in plasma HMGB-1 was positively associated with increased odds of silicosis, and the odds ratio (OR) (95% confidence interval) was 1.86 (1.52, 2.27). Additionally, compared with subjects with lower HMGB-1 concentrations, increased odds of silicosis were observed in those with higher HMGB-1 concentrations, and the OR was 15.33 (6.70, 35.10). Nonlinear models including a natural cubic spline function of continuous HMGB-1 yielded similar results. In ROC analyses, we found that plasma HMGB-1 >7.419 ng/mL had 81.6% sensitivity and 80.4% specificity for silicosis, and the area under the curve (AUC) was 0.84. Our results demonstrated that elevated plasma HMGB-1 was positivity associated with increased OR of silicosis.

## 1. Introduction

Silicosis is a well-known fibrogenic lung disease caused by prolonged inhalation of crystalline silica [1,2]. Characteristic pulmonary tissue pathology in silicosis consists of fibrotic nodules with an arrangement of collagen fibers, central hyalinization, and lesions of massive fibrosis [1,3]. Although the implementation of prevention efforts has been required for decades, silicosis persists worldwide. In developing countries such as China, the incidence of silicosis remains high [1]. According to a report from the Ministry of Health in China, more than 23 million workers are exposed to crystalline silica in China [4], and approximately 10,000 new silicosis cases are diagnosed annually. However, current clinical diagnosis of silicosis mainly depends on chest X-ray and lung function tests, which often revealed abnormal changes in the advanced stage of silicosis [2]. In such a situation, exploring the potential silicosis biomarker for treatment and prevention is urgently needed.

High-mobility group box 1 protein (HMGB-1), a ubiquitous nuclear DNA binding protein, is described as a DNA binding protein that stabilizes nucleosomes and facilitates transcription. HMGB-1 has dual functions. As an intracellular transcription factor, HMGB-1 binds to bent DNA to promote the assembly of nucleoprotein complexes, which is critical in the process of transcription, recombination, replication, and repair. As an extracellular mediator, HMGB-1 acts as a potent inflammatory cytokine [5,6,7]. Previous studies suggest that HMGB-1 is involved in a variety of biological processes and is linked to a wide range of human diseases, such as diabetes [8,9], asthma [10,11], and chronic obstructive pulmonary disease (COPD) [12,13]. Recently, a few experimental studies have provided preliminary evidence that HMGB-1 may play an important role in the inflammation reaction induced by silica exposure. Laurie et al. found that HMGB-1 was upregulated in the macrophages after intratracheally instilling silica dust in mice [14]. Peeters et al. observed a significant dose response relationship between silica uptake and higher expression of HMGB-1 in a human bronchial epithelial cell line [15]. However, epidemiological studies on the relationships between HMGB-1 and silicosis are still scarce.

In the present study, we conducted a control-case study comprising 74 silicosis cases and 107 age/gender-matched healthy controls in China. The objectives of this study were to explore the concentrations of plasma HMGB-1 in healthy controls and silicosis patients, and to investigate the relationships between plasma HMGB-1 levels and silicosis.

## 2. Results

### 2.1. Basic Characteristics of All Subjects

**Figure 1:**
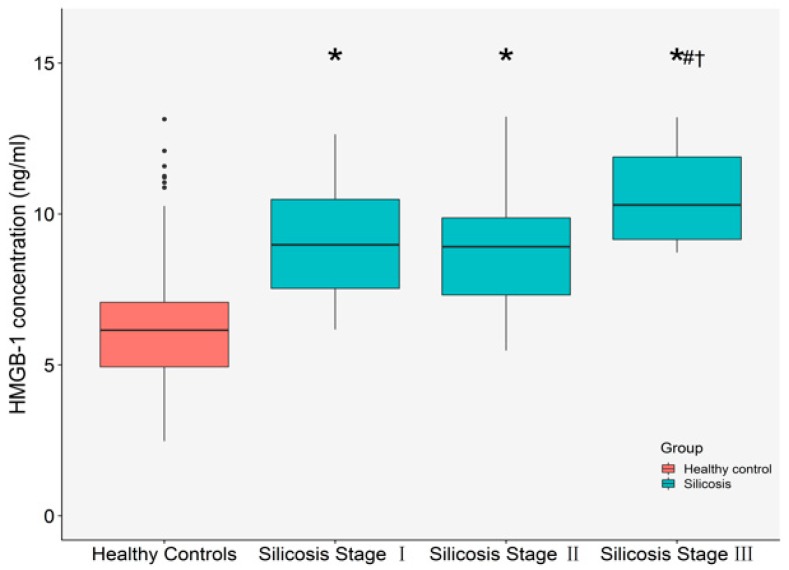
The distributions of plasma HMGB-1 concentrations in different group. * Compared with healthy controls, *p* < 0.05; # compared with stage I of silicosis, *p* < 0.05; † compared with stage II of silicosis, *p* < 0.05. The numbers of healthy controls, silicosis stage I, silicosis stage II, and silicosis stage III were 107, 42, 22 and 10, respectively.

The basic characteristics of all subjects are shown in Table 1. Compared with healthy controls, silicosis patients had remarkably higher plasma concentrations of matrix metalloproteinase-2 (MMP-2), matrix metalloproteinase-9 (MMP-9), collagen alpha-1(I) protein (COL1A1), and collagen alpha-1(III) protein (COL3A1) (all *p* < 0.05). Subjects with silicosis had significant reductions in body mass index (BMI) in comparison with healthy controls. The distributions of plasma HMGB-1 levels are shown in Figure 1. Compared with healthy controls, the concentrations of plasma HMGB-1 were significantly increased in the silicosis (*p* < 0.05). In addition, plasma HMGB-1 levels in stage III were significantly higher than those in the first and the second stages of silicosis (*p* < 0.05).

### 2.2. The Relationships between Plasma HMGB-1 Concentrations and Silicosis

**Figure 2:**
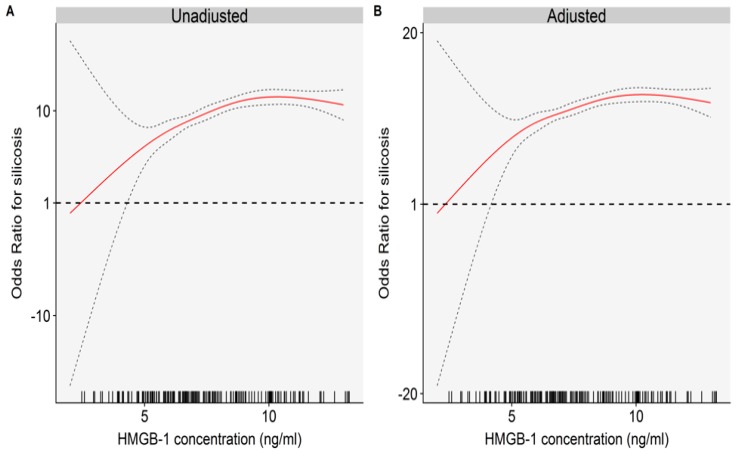
Nonlinear associations between HMGB-1concentrations and silicosis: (**A**) single factor logistic regression; and (**B**) model was adjusted for age (continuous), BMI (continuous), smoking status (no, yes), drinking status (no, yes), and passive smoker (no, yes).

Table 2 presents results of the associations between plasma HMGB-1 levels and silicosis. Each 1 ng/mL increase in plasma HMGB-1 was significantly associated with increased odds of silicosis, and the odds ratio (OR) and (95% confidence interval (CI)) of silicosis was 1.86 (1.52, 2.27) after adjusting the potential confounders. We divided all participants into two groups based on the median of the HMGB-1 concentrations in all subjects. Compared with subjects in the low HMGB-1 levels, the OR of silicosis was 15.33 (6.70, 35.10) in those with higher plasma HMGB-1 after adjusting for potential confounders. Nonlinear models including continuous HMGB-1 as a natural cubic spline function yielded similar results (Figure 2). We further examined the relationships of plasma HMGB-1 with the stage of silicosis. The results showed that ORs of silicosis were significantly increased across the elevated stages of silicosis (Figure S1).

### 2.3. The Discriminatory Power of Plasma HMGB-1 for Silicosis.

**Figure 3:**
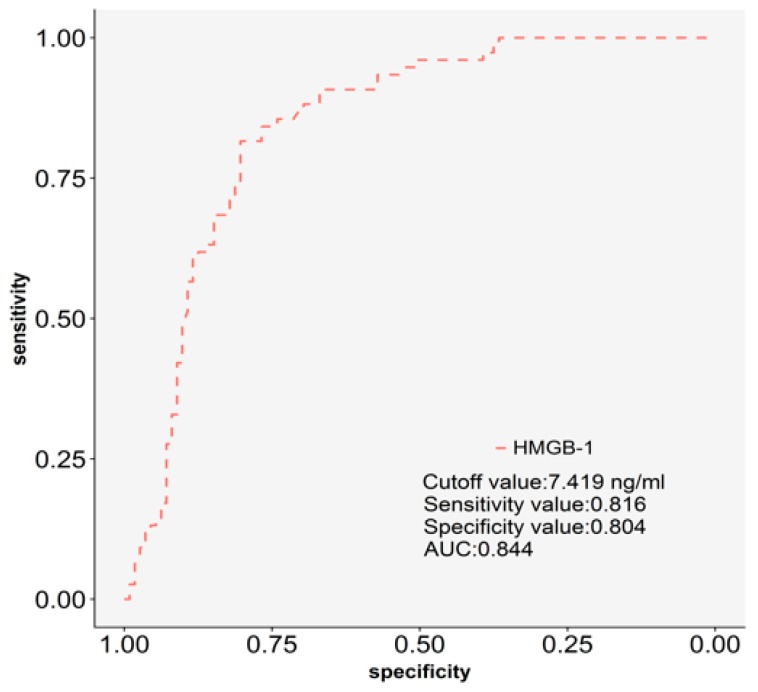
Receiver operating characteristic (ROC) curve analysis for the discriminatory power of plasma HMGB-1 for silicosis.

As shown in Figure 3, we further used ROC curve analysis to examine the discriminatory power of HMGB-1 in plasma for silicosis. Compared with healthy controls, we found that the concentrations of plasma HMGB-1 >7.419 ng/mL had 81.6% sensitivity and 80.4% specificity for detecting silicosis, and the area under the curve (AUC) was 0.84.

### 2.4. The Relationships between Plasma HMGB-1 Concentrations and Clinical/Biological Features in Silicosis Patients

**Figure 4:**
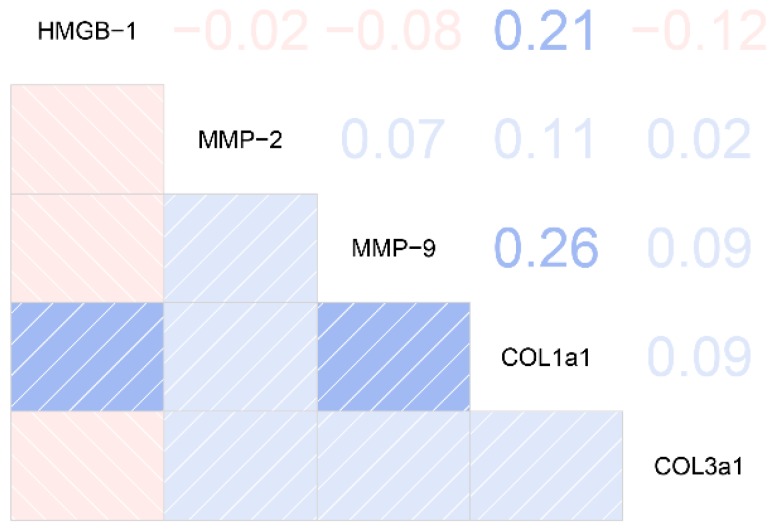
Correlations between plasma HMGB-1 concentrations and clinical/biological features in silicosis patients. Abbreviations: MMP-2, matrix metalloproteinase-2; MMP-9, matrix metalloproteinase-9; COL1A1, collagen alpha-1(I) protein; COL3A1, collagen alpha-1(III) protein.

The relationships between plasma HMGB-1 concentrations and clinical/biological features in silicosis patients are shown in Figure 4. We found that plasma HMGB-1 concentrations were positively correlated with COL1A1 (*r* = 0.21, *p* < 0.05) among silicosis patients.

## 3. Discussion

Our results show that plasma HMGB-1 concentrations were significantly increased in silicosis patients when compared with healthy controls. Elevated plasma HMGB-1 concentrations were positively associated with increased odds of silicosis.

The results of our study have important public health implications. Silicosis continues to be one of the most severe occupational issue worldwide because of its lack of an effective treatment, poor prognosis and late diagnosis. Exploring new biomarkers in the process of silicosis may be an effective means of control and treatment. We found that the elevated plasma HMGB-1 concentrations were positively correlated with increased odds of silicosis. These findings not only indicate that HMGB-1 might serve as a potential biomarker related to silicosis, but also provide novel insights into the inflammatory mechanisms in silicosis. In addition, to our knowledge, this study is the first to report the relationships between plasma HMGB-1 levels and silicosis in humans.

Similar to our results, a few previous in vivo/in vitro studies have demonstrated that silica dust exposure could increase the expression levels of HMGB-1. For instance, Zhang et al. found that SiO_2_ induced a rapid and sustained increase of HMGB-1 expression in RAW264.7 cells [16]. Laurie et al. reported that the expression of HMGB-1 in lung tissues was significantly elevated by intratracheal instillation crystalline silica suspension in mice [14]. Although the above-mentioned studies provide important evidence that long-term silica could induce HMGB-1 overexpression, whether the elevated HMGB-1 also played an important role in the development of silica-induced lung fibrosis is still largely unknown. Previous studies have suggested that HMGB-1 is significantly associated with idiopathic pulmonary fibrosis (IPF). For instance, Hamada et al. reported that the concentrations of HMGB-1 in bronchoalveolar lavage fluid (BALF) were significantly increased in subjects with IPF [17]. Abe et al. found that the concentrations of HMGB-1 in serum were increased in IPF with acute exacerbation [18]. In agreement with previous studies, our results show that the elevated HMGB-1 level was significantly associated with higher odds of silicosis. These results indicate that the HMGB-1-mediated inflammatory signaling pathway might be involved in the process of silicosis.

The pathophysiology behind the relationship between elevated HMGB-1 and silicosis is still unclear and likely complex. Experimental studies have demonstrated that silica exposure induces HMGB-1 overexpression [14,15], which could activate the downstream inflammatory pathway though binding to surface receptors, such as the receptor for advanced glycation end products (RAGE) [7]. The activated signaling pathway not only sustains and amplifies inflammatory response, but also promotes myofibroblast formation and pulmonary fibrosis [19,20,21]. On the other hand, some studies have suggested that the elevated HMGB-1 contributes to pulmonary fibrosis by activating the epithelial-to-mesenchymal transition (EMT) signaling pathway, which might be another route for the development of silica-induced pulmonary fibrosis [22].

Published studies have reported that abnormal HMGB-1 activation is involved in the development of pulmonary fibrosis (PF), and could serve as novel diagnosis or treatment biomarkers for PF. For instance, Chirico et al. found that elevated HMGB-1 was significantly associated with deterioration of lung function and represented as an independent biomarker for monitoring PF [23]. Guiot et al. reported that serum levels of bound HMGB-1 were significantly lower in IPF patients than those in healthy subjects, and the decreased HMGB-1 could serve as a diagnosis and treatment biomarker for IPF [24]. In agreement with the previous studies, we found that HMGB-1 might be a potential biomarker for silicosis. In addition, experimental studies suggest that blocking the HMGB-1/RAGE signaling pathway could reduce collagen protein production (including COL1A1 and COL3A1) and prevent the development of PF [17,23,25]. Therefore, the elevated HMGB-1 and downstream signaling pathway might be a therapeutic target for PF. In our study, we found that the increased HMGB-1 was significantly linked with collagen protein in the silicosis patients. However, to determine whether HMGB-1 could be a new therapeutic or preventive biomarker for silicosis, further studies should be conducted.

Several limitations of this study need to be discussed. First, due to the lack of enrollment of healthy silica-exposed workers, we could not observe changes in HMGB-1 levels at early stages of silicosis and specify whether elevated plasma HMGB-1 concentrations are caused by the silica-dust exposure. Second, we only observed the relationship between HMGB-1 and silicosis, but the underlying mechanisms are still unknown. Third, little is known about the value of HMGB-1 in clinical practice. Further studies are warranted to address the potential role of HMGB-1 in the screening and early diagnosis of silicosis.

In conclusion, we found that HMGB-1 was positivity associated with increased odds of silicosis. Our findings highlight that HMGB-1 may be a potential biomarker for silicosis.

## 4. Materials and Methods

### 4.1. Study Population

A total of 74 male silicosis cases were enrolled from an Occupational Diseases Hospital in Hubei province, central China and 107 age/gender-matched heathy subjects (did not expose to silica) were enrolled from those who had a physical examination at the same hospital. The diagnosis criteria on silicosis in this study were based on the China National Diagnostic Criteria for pneumoconiosis (GBZ 70-2009), which are consistent with the 2000 International Labor Organization on the classification of pneumoconiosis. The criteria describe stages I, II, and III, referring to mild, moderate, and severe silicosis, respectively. Stages I and II correspond to stages 1–2, and 3 in the ILO classification; stage III corresponds to pneumoconiosis with large opacities (categories A, B, and C by the ILO classification). In the present study, we excluded subjects with asthma, pneumonia, COPD, pulmonary tuberculosis and cardiovascular diseases. This research was approved by the Ethics and Human Subject Committees of the Tongji Medical College Huazhong University of Science and Technology (Identification code: (2013) IEC (S017); date: 5 March 2013; Wuhan, China). Written informed consent was obtained from all participants.

### 4.2. Data and Blood Sample Collection

Structured questionnaires were used by trained physicians to collect information on demographic and lifestyle, including date of birth, race, education, smoking status, alcohol consumption status, height, weight, and working history. In addition, we collected a fasting blood sample from each participant and then put into a tube containing EDTA (Ethylenediaminetetraacetic acid). Plasma was obtained by centrifugation at 1500× *g* rpm for 20 min and stored at −80 °C until use.

### 4.3. Covariates

In this study, self-reported education was categorized into college, and higher and lower than college. Individuals who had smoked at least one cigarette per day during the last six months were defined as smokers, and those who had been drank alcohol at least once per week during the last six months were considered drinkers. Body-mass index (BMI, kg/m^2^) was calculated as weight (kg) divided by the square of height (m^2^).

### 4.4. Enzyme-Linked Immunosorbent Assay for Plasma Measurements (ELISA)

Plasma HMGB-1 concentrations were measured using the HMGB-1 ELISA Kit II (Shino Test Corporation, Tokyo, Japan) according to the manufacturer’s protocols. Plasma MMP-2 and MMP-9 concentrations were detected by ELISA commercially available kits purchased from R&D Systems Inc. (Minneapolis, MN, USA), and plasma COL1A1 and COL3A1 concentrations were detected by assay ELISA kits purchased from Uscn Life Science Inc. (Wuhan, China). Each sample was run in duplicate and the mean concentration was determined.

### 4.5. Statistical Analysis

For basic characteristics, continuous variables with normal distribution were presented as means (standard deviation). Differences of basic characteristics were compared using Student’s *t*-test for normal distributed variables and Chi-Squared test for categorical variables. Spearman correlation coefficients were estimated to determine associations between HMGB-1 concentration and clinical/biological features in silicosis. Subjects were divided into two groups (Low/High HMGB-1 concentrations group) based on the median of HMGB-1 concentrations in all subjects. Then, we used multiple logistic regressions to evaluate the associations between HMGB-1 concentrations and the odds of silicosis, with adjustments for age, BMI, smoking status, drink status, passive and smoke status. Furthermore, we conducted a natural cubic spline function of HMGB-1 with four degrees of freedom in the model to identify the dose–response relationship between continuous HMGB-1 and the odds of silicosis. The role of HMGB-1 in the discrimination for silicosis was determined by ROC curve analysis. All analyses were performed using R (The R Foundation for Statistical Computing, Vienna, Austria). All *p* values were two-sided.

## Figures and Tables

**Table 1:** Basic characteristics of study population.

Characteristics	Healthy Subjects (*n* = 107)	Silicosis (*n* = 74)	*p*
Age, years, mean (SD)	59.67 (9.10)	59.48 (9.20)	0.65
BMI, kg/m^2^, mean (SD)	24.69 (3.19)	22.93 (3.16)	<0.05
Current smoking status (*n*, %)			<0.05
Smoker	37 (34.58)	13 (17.57)	
Non-smoker	70 (65.42)	61 (82.43)	
Current drinking status (*n*, %)			0.68
drinker	66 (61.68)	26 (35.14)	
non-drinker	41 (38.32)	48 (64.86)	
Fibrosis-related cytokines, mean (SD)			
MMP-2 (ng/mL)	153.88 (19.35)	203.99 (9.87)	<0.05
MMP-9 (ng/mL)	73.43 (24.02)	124.01 (12.07)	<0.05
COL1A1 (ng/mL)	28.71 (1.42)	34.10 (1.30)	<0.05
COL3A1 (ng/mL)	68.90 (8.50)	112.14 (9.80)	<0.05
Stage of silicosis (*n*, %)			-
Stage I	-	42 (56.75)	
Stage II	-	22 (29.73)	
Stage III	-	10 (13.52)	

Data are given as mean (SD), except that current smoking and drinking status are given as *n* (%). Abbreviations: BMI, body mass index; SD, standardized deviation; MMP-2, matrix metalloproteinase-2; MMP-9, matrix metalloproteinase-9; COL1A1, collagen alpha-1(I) protein; COL3A1, collagen alpha-1(III) protein.

**Table 2:** The relationships between plasma HMGB-1 concentrations and silicosis.

Characteristics	HMGB-1 Concentrations (ng/mL)			
	Per 1 ng/mL Increase of HMGB-1	Low Concentration Group	High Concentration Group	*p*
No. of Case/Control Subjects		11/79	63/28	
Model 1	1.91 (1.57, 2.32)	1.00 (ref.)	16.16 (7.47, 34.97)	<0.05
Model 2	1.89 (1.55, 2.30)	1.00 (ref.)	15.52 (7.10, 33.94)	<0.05
Model 3	1.85 (1.52, 2.26)	1.00 (ref.)	14.52 (6.54, 32.25)	<0.05
Model 4	1.85 (1.52, 2.26)	1.00 (ref.)	14.45 (6.44, 32.43)	<0.05
Model 5	1.86 (1.52, 2.27)	1.00 (ref.)	15.33 (6.70, 35.10)	<0.05

Model 1: single factor logistic regression. Model 2: adjusted for age (continuous). Model 3: adjusted for age (continuous), body mass index (BMI) (continuous). Model 4: adjusted for age (continuous), BMI (continuous), smoking status (no, yes). Model 5: adjusted for age (continuous), BMI (continuous), smoking status (no, yes), drinking status (no, yes), passive smoker (no, yes).

## Supplementary Materials

Supplementary materials can be found at http://www.mdpi.com/1422-0067/19/12/4043/s1.

## Abbreviations

HMGB-1	High-mobility group box-1
ELISA	Enzyme-linked immunosorbent assay
ROC	Receiver operating characteristic curve
OR	Odds ratio
AUC	Area under the curve
COPD	Chronic obstructive pulmonary disease
BMI	Body mass index
SD	Standardized deviation
MMP-2	Matrix metalloproteinase-2
MMP-9	Matrix metalloproteinase-9
COL1A1	Collagen alpha-1(I) protein
COL3A1	Collagen alpha-1(III) protein
CI	Confidence interval
BALF	Bronchoalveolar lavage fluid
RAGE	Receptor for advanced glycation end products
PF	Pulmonary fibrosis
