# Supplementary material for: Association between Plasma HMGB-1 and Silicosis: A Case-Control Study
Source: Int J Mol Sci. 2018 Dec 14;19(12):4043. doi: 10.3390/ijms19124043 (PMC6320808; doi:10.3390/ijms19124043)
Supplement: Supplementary file 1 [file ijms-19-04043-s001.pdf]

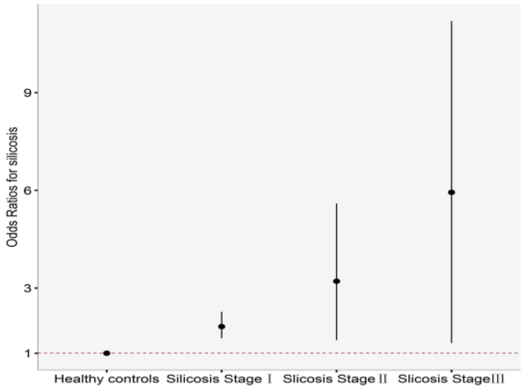

Figure S1 The relationships between HMGB-1 and the stage of silicosis.Model adjusted for age (continuous), BMI (continuous), smoking status (no, yes), drinking status (no, yes), passive smoker (no, yes).
